# Estimating Summer Nutrient Concentrations in Northeastern Lakes from SPARROW Load Predictions and Modeled Lake Depth and Volume

**DOI:** 10.1371/journal.pone.0081457

**Published:** 2013-11-19

**Authors:** W. Bryan Milstead, Jeffrey W. Hollister, Richard B. Moore, Henry A. Walker

**Affiliations:** 1 United States Environmental Protection Agency, Office of Research and Development, National Health and Environmental Effects Research Laboratory, Atlantic Ecology Division, Narragansett, Rhode Island, United States of America; 2 United States Geological Survey, Pembroke, New Hampshire, United States of America; University of Yamanashi, Japan

## Abstract

Global nutrient cycles have been altered by the use of fossil fuels and fertilizers resulting in increases in nutrient loads to aquatic systems. In the United States, excess nutrients have been repeatedly reported as the primary cause of lake water quality impairments. Setting nutrient criteria that are protective of a lakes ecological condition is one common solution; however, the data required to do this are not always easily available. A useful solution for this is to combine available field data (i.e., The United States Environmental Protection Agency (USEPA) National Lake Assessment (NLA)) with average annual nutrient load models (i.e., USGS SPARROW model) to estimate summer concentrations across a large number of lakes. In this paper we use this combined approach and compare the observed total nitrogen (TN) and total phosphorus (TN) concentrations in Northeastern lakes from the 2007 National Lake Assessment to those predicted by the Northeast SPARROW model. We successfully adjusted the SPARROW predictions to the NLA observations with the use of Vollenweider equations, simple input-output models that predict nutrient concentrations in lakes based on nutrient loads and hydraulic residence time. This allows us to better predict summer concentrations of TN and TP in Northeastern lakes and ponds. On average we improved our predicted concentrations of TN and TP with Vollenweider models by 18.7% for nitrogen and 19.0% for phosphorus. These improved predictions are being used in other studies to model ecosystem services (e.g., aesthetics) and dis-services (e.g. cyanobacterial blooms) for ~18,000 lakes in the Northeastern United States.

## Introduction

Global nutrient cycles have been disrupted by the combustion of fossil fuels and the use of fertilizers derived from industrially fixed nitrogen and mined phosphorus [1-6]. A large proportion of this anthropogenic increase in nitrogen and phosphorus flux is delivered to ground or surface waters through direct runoff, human and animal wastes, and atmospheric deposition. Ultimately, excess nutrients are transported to coastal waters [7,8]. 

Increases in nutrient loads to aquatic systems often results in enhanced primary production. This process, known as cultural eutrophication, leads to undesirable changes in aquatic resources such as reduced water clarity, hypoxia, harmful algal blooms, fish kills, loss of biodiversity, and increases in nuisance species [9-11]. Eutrophication can also affect human health through increased exposure to cyanobacteria toxins [12,13], nitrites, and nitrates [14,15]. Furthermore, the economic costs of eutrophication resulting from lost ecosystem services (e.g., housing amenity value, recreation opportunities, freshwater provisioning, and food and fiber production) are high [16-18]. 

In the United States, excess nutrients were reported as the primary cause of lake water quality impairments in the biannual United States Environmental Protection Agency (USEPA) reports to congress from 1994-2002 [19-23]. Given the importance of nutrient pollution, the USEPA [24] requires states to adopt water quality standards with specific numeric nutrient criteria; however, less than half the states have complied [25]. 

The development of nutrient criteria for lakes requires access to reliable information on nitrogen and phosphorus concentrations at the statewide or ecoregion level [24,26]. These data, however, are not always comparable as the field and laboratory methods vary (see 27) often resulting in only a few sites with consistently collected and analyzed nutrient observations . Additionally, differences among sample times can obscure seasonal and inter-annual patterns. In 2007, the USEPA coordinated the National Lake Assessment, a survey of the biological, physical, habitat, and water quality condition for lakes in the 48 contiguous United States [28]. The survey provides consistently collected and analyzed nutrient data for 1152 lakes from the summer of 2007. The majority of the NLA lakes (1028) were selected with a spatially balanced, probabilistic sampling design that was developed to provide inference on the condition of the lakes in the contiguous United States at the national and ecoregional level [28]. An additional set of “hand-selected lakes” were included as reference sites. The National Lake Assessment found that with respect to nutrients approximately half of the lakes are in “Good” condition with the remaining split between “Fair” and “Poor” [28]. Although these data provide useful information on the quality of the nations lakes, the sampling density (mean=21.4 sites/state) is too low for use in the development of nutrient criteria.

An alternative approach is to use models such as the USGS SPARROW (SPAtially Referenced Regression On Watershed attributes) models [29-31] to estimate nutrient loads to lakes. SPARROW models are watershed based models that estimate nutrient loads to streams based on landscape characteristics and known or estimated nutrient sources [30]. An example of this is the Northeast SPARROW model [31]. This model uses an enhanced version of the medium resolution (1:100,000) National Hydrography Dataset (NHDPlus version 1 (V1) [32] to represent the hydrology of the Northeast United States as a network of reaches (called “flowlines” within the NHD, but referred to as “reaches” throughout this article). A reach is a discrete, spatially defined, linear feature located on a hydrologic flow network (i.e., each reach has known upstream and downstream connections) that represents either a stream segment or an artificial path through a waterbody (e.g. wetland, lake, pond, or reservoir). Reaches are associated with unique catchments that include the submerged parts of the local watershed the reach flows through and any land area that drains directly to it.

The Northeast SPARROW model estimates nitrogen and phosphorus contributions to each reach based on 2002 land cover, point source discharges (e.g., waste water treatment facilities), crop types, agricultural fertilizer use levels, animal manure production, and atmospheric deposition (nitrogen only) for the catchment. Not all nutrients that are applied to the land will be delivered to or exported from the streams. Some nutrients will be lost during the land to water delivery phase and others will be lost through instream processes such as nutrient retention. Instream processing varies with stream order and waterbody type [29,31]. The model is calibrated with long-term monitoring data and therefore represents long-term average annual nutrient loads based on 2002 catchment condition. Non-linear least squares regression is used to estimate the model coefficients for source contributions and loss functions that will maximize the fit to the monitoring data [29,31].

In this paper we propose a novel use for the SPARROW model predictions. Although designed to estimate nutrient loads to reaches, the inclusion of waterbody features in both the SPARROW model and NHDplusV1 allow us to aggregate load predictions to lakes. From NHDplusV1 we can identify the stream reaches directly upstream of a lake (the inflows). The sum of the export loads for lake inflows represents the nutrient inputs to the lake from upstream sources. Incremental load (i.e., loads generated within the local catchments before instream processing occurs) predictions for reaches within a lake can be added to the upstream loads to estimate total nutrient inputs. Nutrient loads for all reaches exiting the lake can be summed to predict nutrient exports. 

Moore et al [31] have demonstrated that predicted nutrient concentrations for lakes from the Northeast SPARROW model are consistent with, though higher than values observed during the 2007 National Lake Assessment. This is not surprising since water and nutrient inputs are greatest during the spring runoff period and therefore the average annual predictions of concentration by the SPARROW model will be heavily weighted by spring conditions when nutrient retention within the lake is minimal [33]. Inter-annual variation in precipitation, nutrient inputs, or changes in land cover between 2002 and 2007 will also explain some of the variation. Additionally, estimates of nutrient retention in lakes by the Northeast SPARROW are lower than expected based on a survey of the literature (see below) and this will also affect nutrient load predictions. We hypothesize that the differences between the NLA observations and the SPARROW predictions for TN and TP result from a combination of inter-annual and seasonal variation in water and nutrient inputs coupled with an underestimation of nutrient retention by the model. 

Our overall goal for this paper was to demonstrate that average annual nutrient predictions from SPARROW could be coupled with monitoring data to estimate summer concentrations of nutrients in lakes. The objectives were to (1) adjust the SPARROW predicted annual average concentrations to the observed summer values for 2007 with simple linear models; (2) use Vollenweider type input-output models [35-37] and modeled maximum lake depth and volume [38,39] to improve predictions; and, (3) extrapolate results from the best fit model to the ca. 18000 lakes in the Northeast United States with SPARROW nutrient flux predictions.

## Materials and Methods

### Study Area

**Figure 1:**
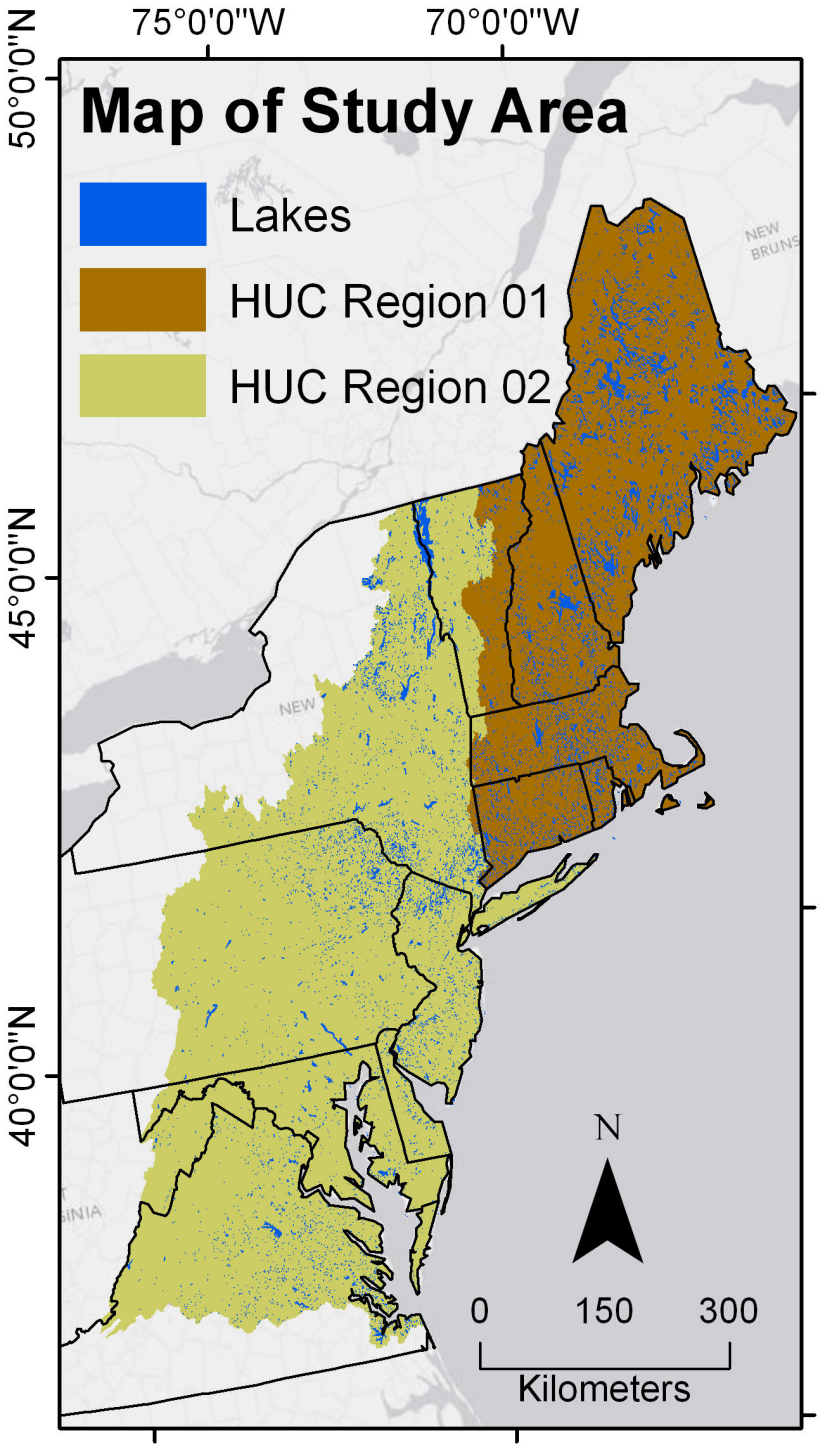
Map of the study area. Shown are the locations of the lakes within Hydrologic Unit Code (HUC) regions 01 (New England) and 02 (Mid-Atlantic).

The study area includes Hydrologic Unit Code regions 01 and 02 from the National Hydrologic Dataset [32], the same areal extent and spatial resolution as the Northeast SPARROW model [31]. This area comprises all or most of Connecticut, Delaware, Maine, Maryland, Massachusetts, New Hampshire, New Jersey, Rhode Island, Vermont, and Washington D.C., much of New York, Pennsylvania, Virginia, and the eastern part of West Virginia (Figure 1). 

### Lake Hydrology, Morphometric, and Water Quality Data

The medium resolution (1:100,000) NHDplusV1 [32], associated tools and value added data tables were used for this analysis. NHDplusV1 for HUC 01 and 02 includes 208,185 reaches and their catchments, and 28,879 waterbody polygons identified as feature type (FTYPE) lake/pond, or reservoir (hereafter lakes). The NHDplusV1 shapefiles and data files were saved in an ESRI personal geodatabase. This allowed the data to be queried as a relational database and geo-processed in ArcGIS (v. 9.2). In NHDplusV1, lakes that intersected the USGS 1:100,000 quad boundaries were split into two or more contiguous polygons. These were re-aggregated into 28,122 uniquely identified (WB_ID) waterbody units by dissolving contiguous polygons of the same feature type in ArcGIS. 

All reaches are assigned a unique identifier (a ComID) by NHDplusV1. Hydrologic relationships among ComIDs are defined in a table included with NHDplusV1 (NHDflow) that identifies the ComIDs of upstream (FromComID) and downstream (ToComID) connections. We used ArcGIS to identify the ComIds of reaches within lakes and these were joined to the table NHDflow in MS Access to identify upstream and downstream connections following the procedures outlined in the NHDplusV1 user guide [40]. When the ComIDs for reaches within a lake are joined to the ToComID in the table “NHDflow” the resulting list of FromComIDs identifies all upstream connections. Since many lakes have multiple internal reaches some of these upstream connections represent connections within the lake and others represent stream segments immediately upstream of the lake (input reaches). Input reaches can be distinguished from internal connections by excluding all FromComIDs that match ComIDs within the lake. Lake outflows, the most downstream reaches within a lake were identified in a similar manner. When the ComIDs for reaches within a lake are joined to the FromComID in table NHDflow the resulting list of ToComIDs identifies all downstream connections. The outflows are those reaches associated with ToComIDs that do not correspond to any of the ComIDs within the lake. 

The surface area (m^2^) of each lake was calculated in ArcGIS version 9.2 following transformation to the Albers equal area projection. Maximum depth (m) and volume (m^2^) were estimated from the surrounding topography from the National Elevation Dataset [41] following the methods of Hollister and Milstead [38] and Hollister et al, [39]. Hydraulic Residence Time (years) was calculated as the ratio of volume to flow. Mean depth (m) was calculated as the ratio of volume to surface area.

The National Lake Assessment water quality data (TN and TP) were obtained from the USEPA [42] and converted to an ESRI personal Geodatabase. Lakes were spatially joined to the NHD waterbodies and assigned the corresponding WB_ID. A total of 131 lakes with NLA data and SPARROW estimates were identified in the Northeast. Of these, 98 lakes were selected for sampling using the probability design and 33 were reference sites. All TN values were above the method detection limit (0.01 mg/l) but 18 of the TP observations were below the detection limit (0.004 mg/l). TP values below the limit were arbitrarily set to 0.002 mg/l for our analysis. 

### Northeast SPARROW Model

The Northeast SPARROW model predictions [31] for total and incremental nitrogen and phosphorus loads (kg/yr) and flow (average annual water inputs [CFS]) were retrieved from the USGS SPARROW Decision Support System [43-45] for all reaches in the study area. Total nitrogen and phosphorus load predictions represent the average annual flux (kg/yr) of nitrogen and phosphorus delivered to the next downstream reach. Total load equals the sum of the nutrient inputs from all reaches immediately upstream of a reach plus the incremental load (load delivered directly to the reach from sources within the local catchment area) minus estimated nutrient decay within the reach itself [29-31]. In this study we used the same logic to calculate nitrogen, phosphorus, and water (flow) inputs and exports for lakes. Upstream inputs of nitrogen and phosphorus were calculated by summing the nitrogen and phosphorus loads for all reaches immediately upstream of the lake. The incremental inputs of nutrients were represented by the sums of the individual incremental nitrogen and phosphorus loads for all reaches within the lake. The total inputs to the lake equal the sums of the upstream inputs and the incremental inputs for each nutrient. The lake nutrient inputs do not include losses due to nutrient retention within the lake and therefore estimate total load before *in situ* processing occurs. Lake exports, the nutrient delivered to the reaches immediately downstream of a lake, are the total load minus in lake nutrient retention. These were calculated by summing the nitrogen and phosphorus loads for the lake outflows. Flow is the average annual input of water to the lake. In the SPARROW model, water is conserved so water exports equal water inputs. Total flow (the average annual input or export of water to the lake) was calculated as the sum of the flows for all lake outflows. Total flow was converted to m^3^/year by multiplying the total CFS by 893,593. Nutrient inputs and exports of nitrogen (N_in_ & N_out_) and phosphorus (P_in_ & P_out_) were converted to concentrations (mg/l) by dividing the total load (kg/yr) by the total flow (m^3^/yr).

The Northeast SPARROW model provided nutrient load predictions for 18,016 of the 28,122 lakes identified in the study area. Lakes without predictions were either small, isolated basins without connections to the larger NHDplusV1 network (no input or output reaches) or were associated with coastal salt pond systems. The SPARROW model estimates a nutrient decay coefficient (based on hydraulic load for lakes) from the data but the decay term is only included if it is statistically significant. For reaches in lakes the Northeast SPARROW model found a significant decay coefficient for phosphorus but not nitrogen [31]. As a result, nitrogen inputs to lakes should equal exports unless there are hydrological issues such as water diversions within or immediately upstream of the lakes. A total of 17,792 lakes met this conservation of nitrogen mass balance criterion (N_in_ = N_out_) for inclusion in this study.

### Vollenweider Models

Vollenweider models (input-output models) are used to predict lake nutrient concentrations from nutrient inputs, residence time, and (sometimes) mean depth [34-36]. Brett and Benjamin [35] identified five input-output models variations (H_1_ to H_5_ in [Table pone-0081457-t001]) used to estimate lake phosphorus concentrations from phosphorus input concentrations. Brett and Benjamin [35] compared the five models to the null hypothesis (H_0_: lake concentration = output concentration) and found H_4_ to be best supported. A sixth variation (H_6_; Table 1) was used by Reckhow [36] to estimate phosphorus concentrations for the EUTROMOD model. The majority of the input-output models in the literature have been used to predict phosphorus concentrations in lakes. However, Vollenweider [34] applied his original input-output models to both nitrogen and phosphorus, Bachman [46] and Reckhow [36] used a variation of H_4_ for nitrogen, and Windolf et al [37] developed two input-output model variations for nitrogen (see H_7_ and H_8_; Table 1). The input-output models are simple mass balance equations that estimate the concentration of chemical substances based on inputs, outputs and sedimentation [34]. Therefore they are equally applicable to the estimation of TN and TP concentrations. To test whether an independent estimate of nutrient retention improves our ability to predict nutrient concentrations we used the eight input-output models in Table 1 to predict nitrogen and phosphorus concentrations for the Northeast lakes. The input-models were parameterized with the SPARROW predicted nutrient input concentrations (N_in_ & P_in_) and our estimates of hydraulic residence time and mean depth; the NLA observed TN and TP concentrations were used to validate the models. 

**Table 1 pone-0081457-t001:** Hypotheses tested.

**Hypothesis**	**Model**	**Reference**
H_0_	$\log_{10}(Nutrient_{lake})=\log_{10}(Nutrient_{out})$	[35]
H_1_	$\log_{10}(Nutrient_{lake})=\log_{10}(\frac{Nutrient_{in}}{1+0.45\tau })$	[35]
H_2_	$\log_{10}(Nutrient_{lake})=\log_{10}(\frac{Nutrient_{in}}{1+1.06})$	[35]
H_3_	$\log_{10}(Nutrient_{lake})=\log_{10}(\frac{Nutrient_{in}}{1+\frac{5.1}{z}\tau })$	[35]
H_4_	$\log_{10}(Nutrient_{lake})=\log_{10}(\frac{Nutrient_{in}}{1+1.12\tau^{0.53}})$	[35]
H_5_	$\log_{10}(Nutrient_{lake})=\log_{10}(\frac{0.65Nutrient_{in}}{1+0.17\tau })$	[35]
H_6_	$\log_{10}(Nutrient_{lake})=\log_{10}(\frac{Nutrient_{in}}{1+3.0\tau^{0.25}z^{0.58}Nutrient_{in}{}^{0.53}})$	[36]
H_7_	$\log_{10}(Nutrient_{lake})=\log_{10}(0.32Nutrient_{in}\tau^{-0.18})$	[37]
H_8_	$\log_{10}(Nutrient_{lake})=\log_{10}(0.27Nutrient_{in}\tau^{-0.22}z^{-0.12})$	[37]

H_0_ is the null hypothesis that lake concentration of nutrients (nitrogen and phosphorus) is equal to the export concentration for reaches leaving the lake. H_1_-H_8_ are the Vollenweider (input-output) models with parameters and initial coefficients taken from the literature used to fit the models. Note: H_1_-H_6_ were originally derived to estimate total phosphorus and H_7_ & H_8_ were derived to estimate total nitrogen. Nutrient_lake_ = Measured nutrient (nitrogen or phosphorus) concentration (mg/l) for the lake. Nutrient_in_ SPARROW predicted average annual nutrient (nitrogen or phosphorus) input concentration (mg/l) for the lake; Nutrient_out_ = SPARROW predicted average annual nutrient (nitrogen or phosphorus) concentration (mg/l) for the lake; z = mean depth (m); and, τ = hydraulic residence time (years).

### Statistical Analysis

**Figure 2:**
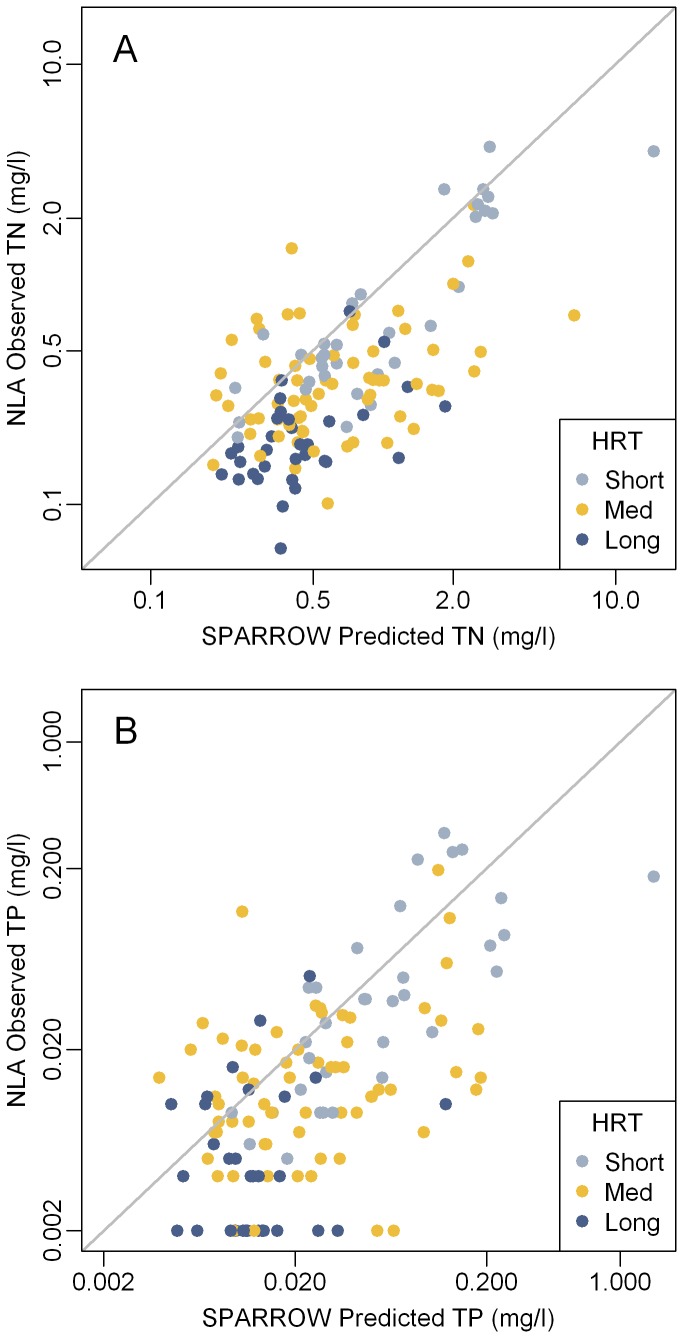
Total nitrogen (A) and phosphorus (B) in Northeast Lakes. National Lake Assessment 2007 observed summer concentrations versus the average annual SPARROW predicted concentrations. Observations are color coded by hydraulic residence time (HRT: Short < 0.04 years; Medium = 0.04 to 0.4 years; Long > 0.4 years). TN = Total Nitrogen. TP = Total Phosphorus.

**Figure 3:**
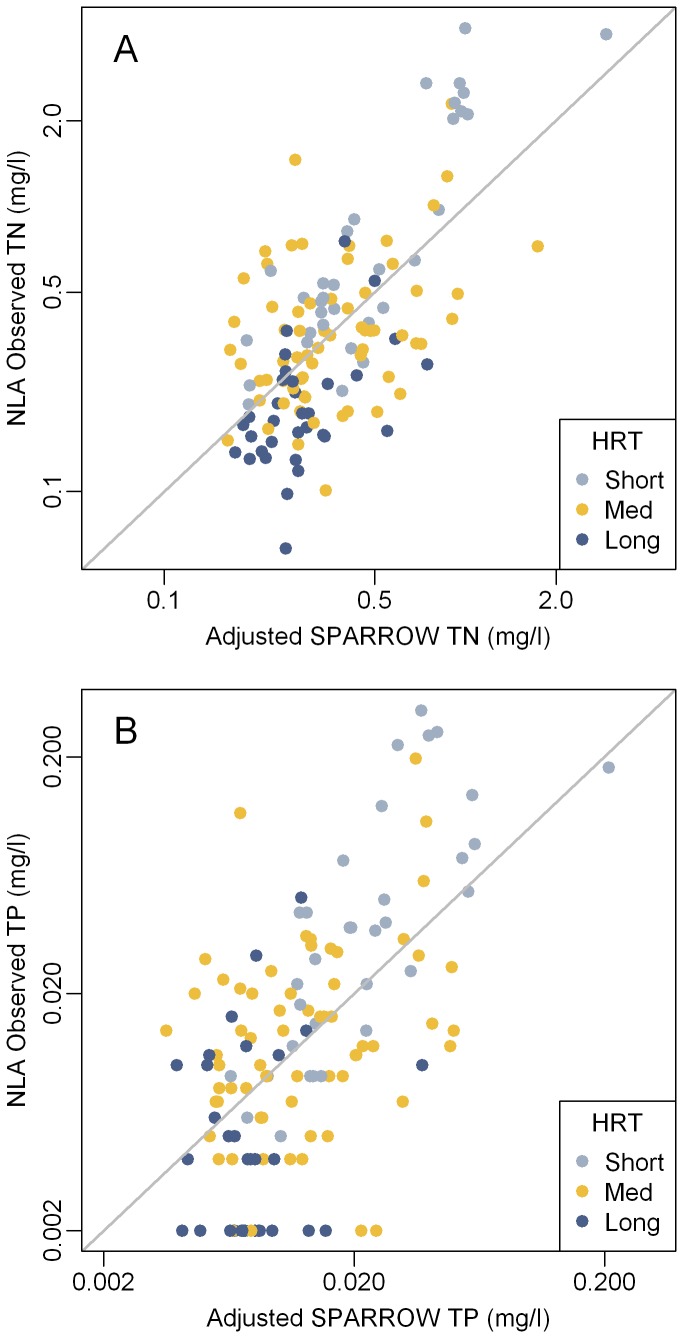
Adjusted (Linear Model) total nitrogen (A) and phosphorus (B) in Northeast Lakes. National Lake Assessment observed 2007 summer concentrations of (A) total nitrogen and (B) phosphorus in Northeast Lakes versus the linear model (LM) adjusted average annual SPARROW predicted concentrations. Linear regression was used to adjust SPARROW predictions to the 2007 NLA observations. Observations are color coded by hydraulic residence time (HRT: Short < 0.04 years; Medium = 0.04 to 0.4 years; Long > 0.4 years). TN = Total Nitrogen. TP = Total Phosphorus.

**Figure 4:**
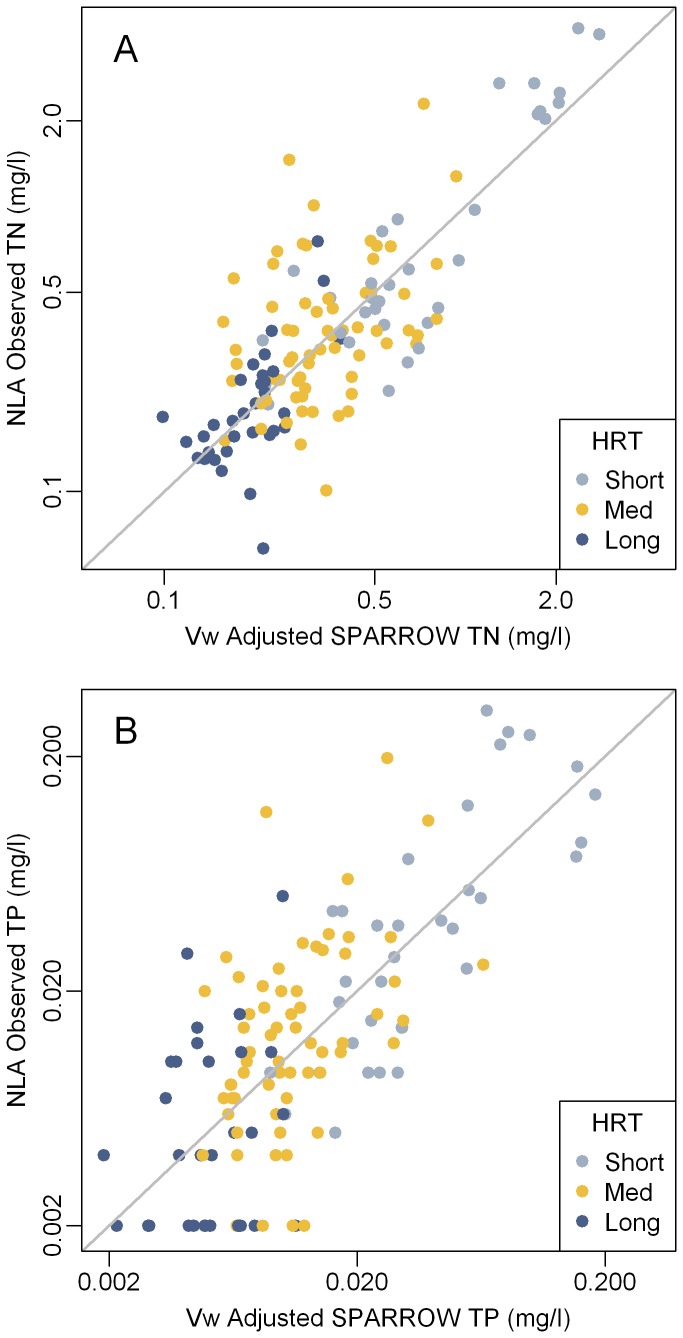
Adjusted (Vollenweider Model) total nitrogen (A) and phosphorus (B) in Northeast Lakes. National Lake Assessment observed 2007 summer concentrations of (A) total nitrogen and (B) phosphorus in Northeast Lakes versus the Vollenweider (Vw) adjusted average annual SPARROW predicted concentrations. Robust non-linear regression was used to fit SPARROW predictions to 2007 NLA observations using the Vollenweider equation (H_6_). Observations are color coded by hydraulic residence time (HRT: Short < 0.04 years; Medium = 0.04 to 0.4 years; Long > 0.4 years). TN = Total Nitrogen. TP = Total Phosphorus.

**Figure 5:**
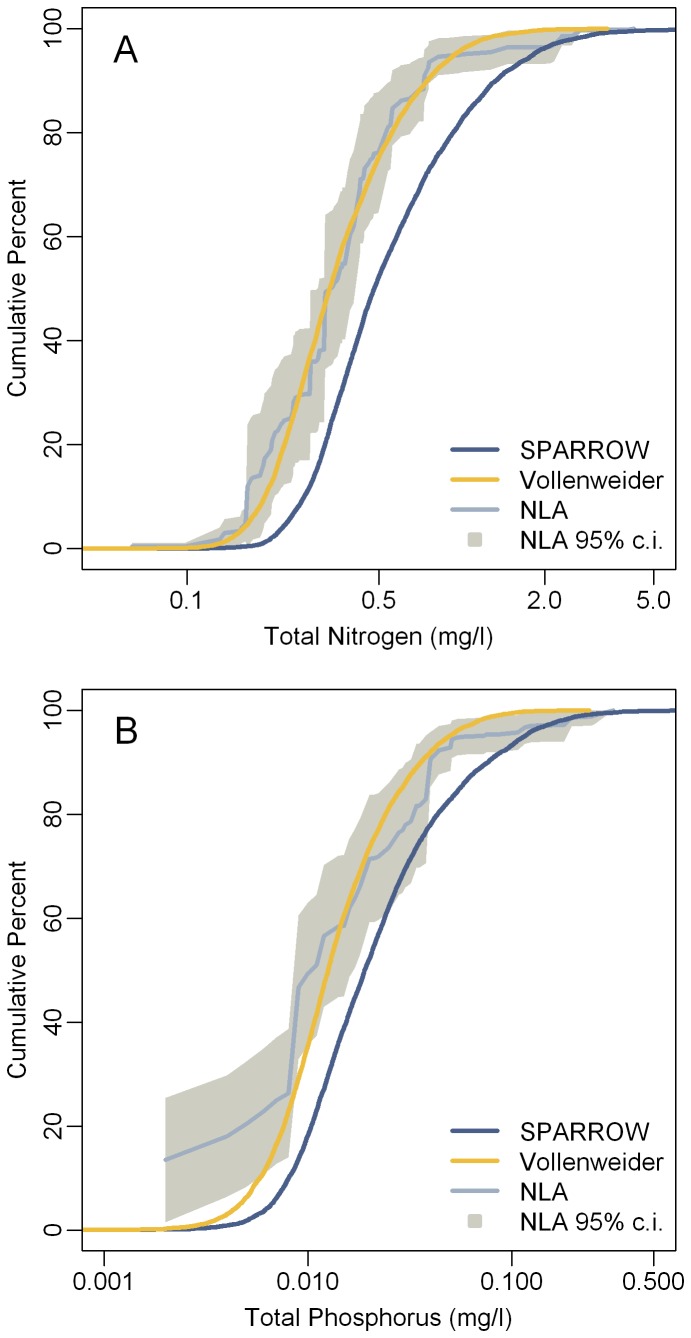
Cumulative distribution functions for observed and predicted nitrogen (A) and phosphorus (B) concentrations. Cumulative distribution functions for SPARROW predictions, the Vollenweider (H_6_) adjusted SPARROW predictions, and the 2007 National Lake Assessment observations. The grey polygons represent the weighted 95% confidence intervals for the NLA cumulative distributions. Note: Only the SPARROW predictions consistent with the NLA sampling design (area ≥ 4 ha; maximum depth ≥ 1 m; n=7669) and the NLA lakes selected under the probabilistic design were included (n=98).

**Figure 6:**
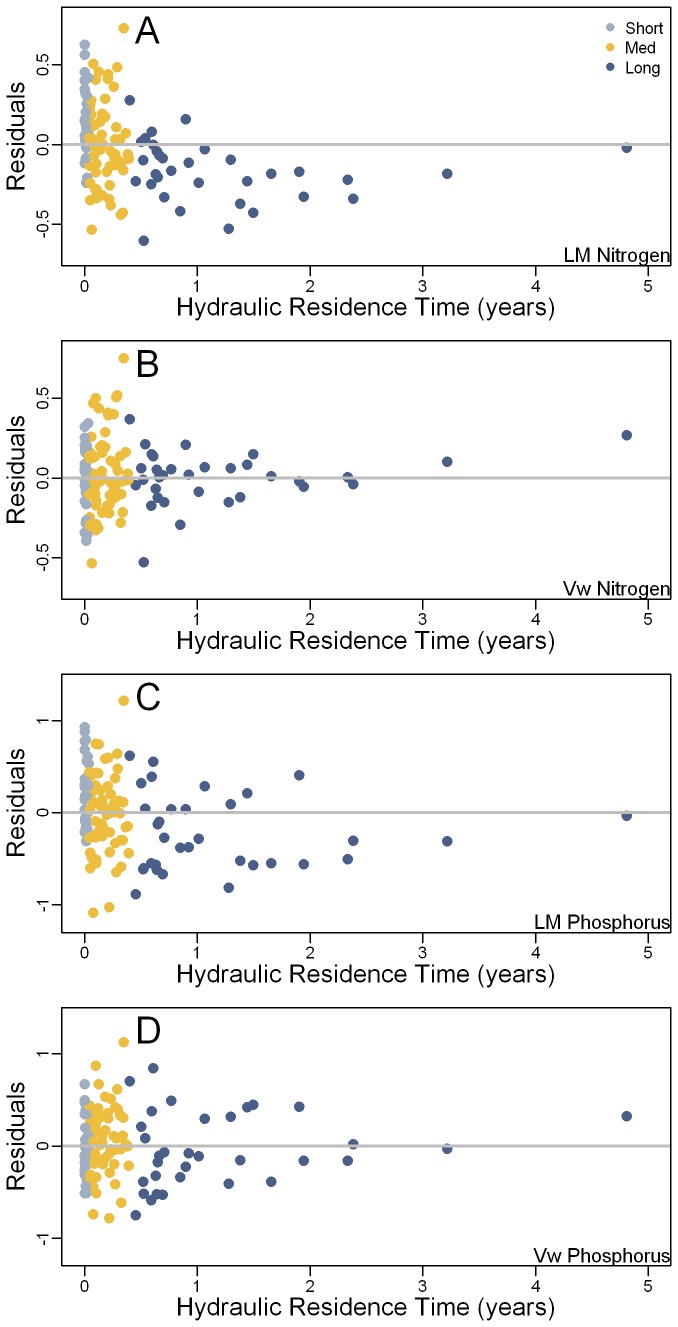
Plot of model residuals by hydraulic residence time. Panels: (A) linear model (LM) for total nitrogen; (B) Vollenweider (Vw) model for total nitrogen; (C) linear model (LM) for total phosphorus; and, (D) Vollenweider (Vw) model for total phosphorus. For both nutrients, the linear models (A and C) show a bias towards negative residuals (overestimation) for longer residence times. In contrast, the residuals for the Vollenweider models (B and D) are independent of residence time. Observations are color coded by hydraulic residence time (Short: < 0.04 years; Medium: 0.04 to 0.4 years; Long: > 0.4 years).

**Figure 7:**
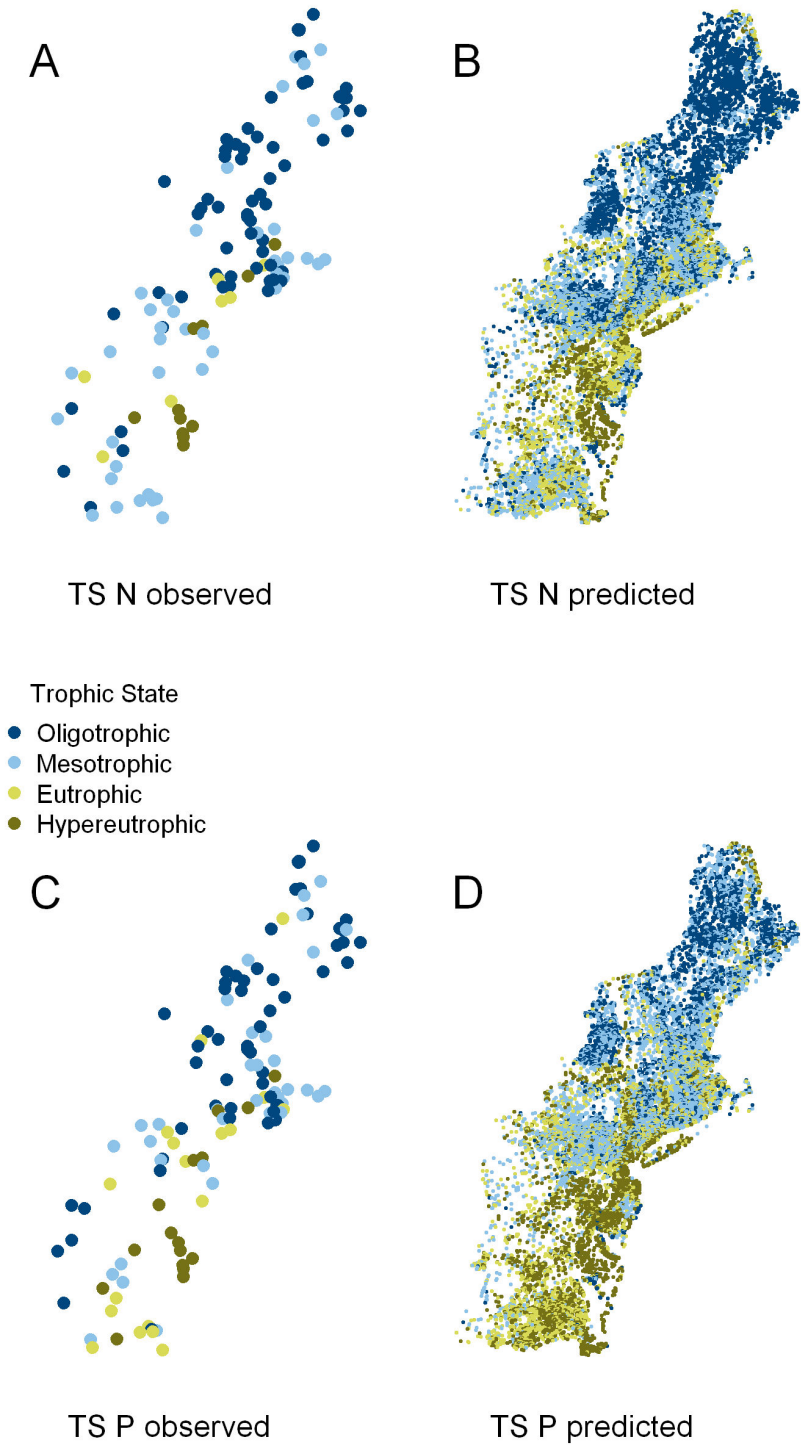
The geographical distribution of the Northeast lakes coded by trophic state. The National Lake Assessment Lake centroids with trophic state (TS) estimated from observed total (A) Nitrogen and (B) Phosphorus concentrations. Lakes with trophic state estimated from the Vollenweider adjusted predicted total (C) nitrogen and (D) phosphorus concentrations.

All analyses were completed with the open source statistical package R version 2.14.2 [47]. The R package “spsurvey” [48] was used to calculate confidence intervals for the NLA observations. The statistical analyses, tables, and figures 2-7 can be reproduced with the R-script (Text S1) and the R-dataset (Dataset S1) included as supplements to this publication. 

Our null hypothesis (H_0_; Table 1) was that the NLA observed in-lake nutrient concentrations could be estimated from the SPARROW predicted export load concentrations (N_out_ & P_out_). The expectation was that the observed values would differ from the SPARROW predictions due to seasonal and inter-annual variation in inputs but that the variation could be reduced by fitting the data to a simple linear model. For NLA lakes the unadjusted SPARROW predictions were fit to observed values with linear regression (lm procedure in R; Nutrient_obs_ ~ Nutrient_out_); all variables were log_10_ transformed. The resulting model was used to produce new estimates of nutrient concentrations for the NLA lakes. 

To test whether the SPARROW predictions could be improved by the application of input-output models we followed a two step procedure. First, we used robust non-linear regression (nlrob procedure from the R robustbase package [49]) to fit the observed NLA TN and TP concentrations, predicted SPARROW nutrient input concentrations (N_in_ & P_in_), and the estimated hydraulic residence times and mean depths to the eight Vollenweider models listed in Table 1. Second, for each nutrient the input-output model with the lowest AIC (Akaike Information Criterion) value was selected and used to generate new nutrient concentration predictions for the NLA lakes.

Three estimates of TN and TP concentrations were made for each NLA lake; the uncorrected SPARROW output predictions (N_out_ & P_out_), linear model adjustments to N_out_ and P_out_, and the predictions from the best fit input-output models. Each estimate was compared to the observed values with linear regression and the adjusted R^2^ was used as an estimate of the amount of variance explained by the model. Root mean squared error (RMSE) was used as an estimate of goodness of fit. The model with the lowest RMSE and highest adjusted R^2^ was selected and used to predict the nutrient concentrations of the 17,792 lakes in the region with useable SPARROW information.

### Permits

No permits were required for the described study, which complied with all relevant regulations.

## Results and Discussion

We successful accomplished the three objectives of this paper to: (1) adjust the SPARROW predicted annual average concentrations to the observed summer values for 2007 with simple linear models; (2) use Vollenweider type input-output models [35-37] and modeled maximum lake depth and volume [38,39] to improve predictions; and, (3) extrapolate results from the best fit model to the ca. 18000 lakes in the Northeast United States with SPARROW nutrient flux predictions.

### Comparison of SPARROW to the National Lakes Assessment

The relationship between observed and predicted nutrient concentration for both nitrogen and phosphorus appear to be linear (Figure 2). However, for the 131 lakes with both NLA observations and SPARROW predictions 76% of the TP estimates and 83% of the TN estimates fall below the one-to-one line (Figure 2) indicating the SPARROW predicts higher nutrient concentrations than were observed during the summer of 2007. 

A large proportion of these differences are likely due to a combination of inter-annual and seasonal variation in nutrient loads along with model and sampling error. SPARROW estimates are based on long-term annual means at monitored sites. Therefore, observations from any given year will differ from the SPARROW estimates due to fluctuations in inputs, flow, and climatic conditions. In the Northeast U.S. most of the annual nutrient flux occurs following snow melt in the spring [33]. Therefore, summer nutrient concentrations are expected to be lower than the annual means [33] since inputs are lowest and aquatic decay is greatest during the summer months. Based on the assumption that these changes are both linear and additive, linear regression was used to adjust the predicted 2002 mean annual nutrient concentrations from the SPARROW model to the 2007 NLA observed summer conditions. This approach (H_0_) explains 43% and 35% of the variation in TN and TP concentrations respectively (Table 2 and Figure 3). These results indicate the SPARROW model can be used to estimate nutrient concentrations with a reasonable level of accuracy. Similar results are also reported in Moore et al, [31]. 

**Table 2:** Model selection results by hypothesis and nutrient.

	**Nitrogen Results**			**Phosphorus Results**		
**Hypothesis**	**rmse**	**adjR2**	**aic**	**rmse**	**adjR2**	**Aic**
H_0_	0.26	0.43	24.7	0.43	0.35	154.3
H_1_	0.31	0.53	21.4	0.47	0.45	141.9
H_2_	0.28	0.43	8.7	0.46	0.30	152.7
H_3_	0.31	0.48	6.2	0.48	0.38	158.2
H_4_	0.27	0.51	-8.4	0.44	0.42	141.6
H_5_	0.28	0.49	4.0	0.44	0.40	140.2
H_6_	**0.21**	**0.62**	**-69.9**	**0.36**	**0.54**	**94.1**
H_7_	0.27	0.52	-5.1	0.44	0.41	142.0
H_8_	0.27	0.52	-4.6	0.44	0.42	142.3

Hypothesis H_6_ (in bold) had the lowest aic and rmse values for both nutrients. Abbreviations: rmse = the root mean squared error; adjR^2^ = coefficient of determination adjusted for number of estimated parameters; aic = Akaike information criterion.

### Improved Predictions with Vollenweider Models

An important source of bias became evident when nutrient concentration data are coded by hydraulic residence time (Figures 2 and 3). Estimated nutrient concentrations for lakes with long residence times (>0.4 years; the fourth quartile for NLA lakes) tended to be higher than observed values while the opposite trend occurred in lakes with short residence times (<0.04 years; the first quartile for NLA lakes). For both nutrients, the means of the model residuals were significantly different (p < 0.0001) between lakes with long and short residence times (Table 3) thus confirming a prediction bias due to residence time. To adjust for this, the data were fit to Vollenweider type input-output models H_1_-H_8_ (Table 1) by robust non-linear regression. Most of the models improved the prediction accuracy of the SPARROW model (Table 2). Based on AIC, H_6_ was selected as the best model for both phosphorus and nitrogen. Use of this model explained substantially more of the variation in observed vs. predicted concentration for both nutrients than the linear model alone. The adjusted R^2^ values for TN improved from 0.431 for the linear model to 0.618 for the Vollenweider model (H_6_) and the root mean squared error (RMSE) decreased from 0.260 to 0.214 (Table 2). For TP, H_6_ resulted in a higher adjusted R^2^ (0.541) and lower RMSE (0.359) than the linear model (adjusted R^2^ = 0.351; RMSE = 0.426; Table 2). The final parameterized Vollenweider models (H_6_) with calibrated coefficients are shown in Equations 1 and 2 (N_lake_ and P_lake_ = lake TN and TP concentrations [mg/l]; N_in_ and P_in_ = TN and TP input concentrations [mg/l]; τ = hydraulic residence time [years]; and, z = mean depth [m]).

**Table 3:** T-test of residual means by hydraulic residence time for each nutrient.

**Hypothesis**	**Nutrient**	**HRT**	**Mean**	**SD**	**N**	**t**	**d.f**	**P**
H_0_	Nitrogen	Short	0.171	0.210	33	6.91	63.4	<0.001
		Long	-0.169	0.190	33			
	Phosphorus	Short	0.218	0.340	33	4.70	61.8	<0.001
		Long	-0.220	0.413	33			
H_6_	Nitrogen	Short	0.002	0.188	33	-0.07	63.1	0.941
		Long	0.005	0.166	33			
	Phosphorus	Short	-0.010	0.317	33	0.26	61.0	0.796
		Long	-0.033	0.399	33			

Residuals from the linear and Vollenweider models for lakes with short (< 0.04 years) and long (> 0.4 years) residence times were compared with a t-test of the means. SD = standard deviation. N=number of observations by group. t = Student’s t-statistic. d.f. = degrees of freedom. P = probability.

. 

$$\log_{10}(N_{lake})=\log_{10}(\frac{N_{in}}{1+2.0\tau^{0.38}z^{0.29}N_{in}{}^{1.14}})$$ Equation 1

$$\log_{10}(P_{lake})=\log_{10}(\frac{P_{in}}{1+89.0\tau^{0.40}z^{0.57}P_{in}{}^{1.08}})$$ Equation 2

Use of the Vollenweider model H_6_ improves estimates of summer nutrient levels (Figure 4). Cumulative distribution functions and their 95% confidence intervals were calculated for the observed NLA concentrations of TN and TP with the R package “spsurvey” [48]. These were compared to the TN and TP concentrations predicted by SPARROW and the Vollenweider adjusted SPARROW predictions. For both nitrogen and phosphorus the Vollenweider adjusted predictions closely approximated the observed distribution (within the 95% confidence interval) whereas the unadjusted SPARROW predictions did not (Figure 5). 

More importantly, however, the Vollenweider model decreased the bias associated with residence time. When the Vollenweider predictions were compared to the observed summer values the predictions were symmetrical around the one-to-one line (Figure 4). The differences between the linear models and the Vollenweider models are apparent when the model residuals are plotted against residence time (Figure 6). For the linear models there is a clear change from under prediction to over-prediction as residence time increases, whereas the Vollenweider residuals for both nutrients are symmetrical around the zero line. Overall the residuals for the Vollenweider models showed less deviation than those for the linear model and there were no significant differences (p > 0.05) in means of the residuals for lakes with long and short residence times (Table 3). This highlights the importance of accounting for both nutrient inputs and residence time when estimating nutrient concentrations in lakes. Whereas both the linear model and the non-linear Vollenweider model adjust the model results to summer 2007 conditions, only the Vollenweider adjusted estimates control for differences in nutrient retention related to hydraulic residence time.

The Northeast SPARROW model predicts no nitrogen retention (100*[Input- Output] / Input) and low phosphorus retention (median = 8.3%; mean= 14.0%; s.d. = 15.40) for the Northeast Lakes. These nutrient retention predictions are low compared to other published studies. Saunders and Kalff [50] found on average lakes retain 34% percent of nitrogen and similar values for nitrogen retention are reported by Reckhow ([36]; mean=35%), Windolf et al, ([37]; mean=33%), and Harrison et al, ([51]; reported as ranges). In an analysis of data from 305 lakes Brett and Benjamin [35] report a mean phosphorus retention of 40% (median = 45%) which is consistent with the results of Hejzlar et al, ([52]; lake mean = 46%; reservoir mean = 43%) but lower than the 60% reported by Reckhow [36]. When compared to the SPARROW model the H_6_ Vollenweider models show much higher levels of retention for both nitrogen (median = 20.7%; mean= 24.6%; s.d. = 18.51) and phosphorus (median = 29.5%; mean= 33.5%; s.d. = 25.17). Caution needs to be exercised in interpreting the nutrient retention calculations from H_6_ because they are confounded with the corrections for seasonal and annual variation in inputs. However, the reductions in bias related to residence time suggest that the Vollenweider model give a more accurate representation of nutrient retention than the SPARROW model alone.

The Northeast SPARROW model estimates nutrient inputs, nutrient retention, and land to water delivery fractions directly from the data [31]. It is possible to include a user defined nutrient retention estimate in the SPARROW model and this approach has been used successfully by Alexander et al, [53]. Our results suggest that incorporating a user defined retention estimate or a nutrient loss function based on hydraulic residence time for the reaches in lakes will improve the fit for the Northeast SPARROW model.

### Predicting Summer Nutrient Concentration in Northeastern Lakes

The Vollenweider adjusted SPARROW predictions provide reasonable estimates for the TN and TP concentrations observed during the 2007 National Lake Assessment. Equations 1 and 2 are used to extend these predictions to 17,810 lakes in the Northeast region of the United States. To visualize the data, we follow the 2007 National Lake assessment [28] in assigning trophic status to lakes as follows: oligotrophic (TN ≤ 0.35 mg/l; TP ≤ 0.01), mesotrophic (0.35 < TN ≤ 0.75 mg/l; 0.01 < TP ≤ 0.25), eutrophic (0.75 < TN ≤ 1.4 mg/l; 0.25 < TP ≤ 0. 5), and hypereutrophic (TN > 0.5 mg/l; TP > 1.4). Figure 7 shows the trophic status of Northeastern lakes in based on observed (NLA) and predicted (Vollenweider adjusted) nutrient concentrations. Visually, there is high degree of spatial concordance between observed and predicted trophic state with similar patterns for both nutrients. The trend is for a predominance of lower nutrient, oligotrophic and mesotrophic lakes in the north and higher elevation sites in the south. In contrast, higher nutrient, eutrophic and hypereutrophic lakes are more common in the agricultural areas of the Chesapeake drainage and the urbanized areas of the mid-Atlantic region. 

### Potential Use of Predicted Nutrient Concentrations

In this paper we demonstrate how the predictions from the USGS SPARROW model can be used to assess summer nutrient concentrations in lakes. Although the SPARROW model was designed to give reach level information for streams, reaches within lakes can be aggregated to estimate long-term flow-weighted average annual nutrient concentrations in lakes. These concentrations, however, may not reflect the summer conditions that are of greater interest to lake managers. Furthermore, average annual conditions may not accurately capture inter-annual variation in inputs. By fitting Vollenweider models to monitoring data the SPARROW predictions can be used to more accurately predict summer nutrient concentrations in lakes. Care should be used in interpreting the results. The Northeast SPARROW model is based on 2002 landscape conditions and it is highly likely that for any given watershed these conditions will have changed. Whereas the prediction uncertainty may be high for individual lakes, the SPARROW model gives a reasonable assessment of conditions for lakes aggregated at the state and regional levels. 

The modified SPARROW predictions for nutrient concentrations in lakes could be useful to States for the design of monitoring programs aimed at the development of water quality standards and the assessment of impaired waters under the section 303d of the Clean Water Act. Although modeled nutrient concentrations will never replace monitoring data, they could be used to target limited sampling funds to areas with highest estimated nutrient concentrations or greatest uncertainties [54]. 

Once impairments have been established, the SPARROW model predictions could also be used as a tool to evaluate scenarios for TMDL (total maximum daily load) reductions necessary to remove impaired lakes from the 303d list. In addition to predicting total loads, the SPARROW model also provides numerical estimates of loads by sources such as agriculture, atmospheric, deposition, and runoff from urban areas (see 31). The USGS has recently released a web-based SPARROW decision support system that allows managers to estimate changes in loads that will accrue from modifications of nutrient input sources [43]. This tool could be used to predict how changes in management practices will affect nutrient loads to streams and lakes. 

Many ecosystem services, such as lake shore housing amenity value, recreational opportunities for fishing, wildlife viewing, boating, contemplation, and the provisioning of safe drinking and irrigation water, are stongly affected by nutrient loads. Ecosystem dis-services such as cyanobacteria and their human and animal health risks are also affected by nutrients in lakes. As a result, it will be possible to also use the SPARROW decision support system to model how changes to loads could affect ecosystem services in lakes. 

## Supporting Information

Dataset S1 **Nitrogen, phosphorus, flow, and lake morphometry data used in the analyses.** See Text S1 for data definitions. Data in comma separated value (CSV) format.
(CSV)

Text S1 **R-code in text format to replicate the analyses.** Use this code in conjunction with Dataset S1 to replicate the statistical analyses, tables and figures 2-7.
(TXT)
